# A Novel Scheme for Full Bottom Dielectric Isolation in Stacked Si Nanosheet Gate-All-Around Transistors

**DOI:** 10.3390/mi14061107

**Published:** 2023-05-24

**Authors:** Jingwen Yang, Ziqiang Huang, Dawei Wang, Tao Liu, Xin Sun, Lewen Qian, Zhecheng Pan, Saisheng Xu, Chen Wang, Chunlei Wu, Min Xu, David Wei Zhang

**Affiliations:** 1 School of Microelectronics, Fudan University, Shanghai 200433, China; 18112020011@fudan.edu.cn (J.Y.); 20212020016@fudan.edu.cn (Z.H.); 20112020120@fudan.edu.cn (D.W.); 20112020027@fudan.edu.cn (X.S.); 21112020140@m.fudan.edu.cn (L.Q.); 20212020008@fudan.edu.cn (Z.P.); ssxu@fudan.edu.cn (S.X.); chen_w@fudan.edu.cn (C.W.); wuchunlei@fudan.edu.cn (C.W.); 2 Shanghai Integrated Circuit Manufacturing Innovation Center Co., Ltd., Shanghai 201203, China

**Keywords:** Si nanosheet gate-all-around (NS-GAA) transistors, S/D-first, fully bottom dielectric isolation (BDI), Full BDI_Last, TCAD simulation

## Abstract

In this paper, a novel scheme for source/drain-first (S/D-first) full bottom dielectric isolation (BDI), i.e., Full BDI_Last, with integration of a sacrificial Si_0.5_Ge_0.5_ layer was proposed and demonstrated in a stacked Si nanosheet gate-all-around (NS-GAA) device structure using TCAD simulations. The proposed full BDI scheme flow is compatible with the main process flow of NS-GAA transistor fabrication and provides a large window for process fluctuations, such as the thickness of the S/D recess. It is an ingenious solution to insert the dielectric material under the source, drain and gate regions to remove the parasitic channel. Moreover, because the S/D-first scheme decreases the problem of high-quality S/D epitaxy, the innovative fabrication scheme introduces full BDI formation after S/D epitaxy to mitigate the difficulty of providing stress engineering in the full BDI formation before S/D epitaxy (Full BDI_First). The electrical performance of Full BDI_Last is demonstrated by a 4.78-fold increase in the drive current compared to Full BDI_First. Furthermore, compared to traditional punch through stoppers (PTSs), the proposed Full BDI_Last technology could potentially provide an improved short channel behavior and good immunity against parasitic gate capacitance in NS-GAA devices. For the assessed inverter ring oscillator (RO), applying the Full BDI_Last scheme allows the operating speed to be increased by 15.2% and 6.2% at the same power, or alternatively enables an 18.9% and 6.8% lower power consumption at the same speed compared with the PTS and Full BDI_First schemes, respectively. The observations confirm that the novel Full BDI_Last scheme incorporated into an NS-GAA device can be utilized to enable superior characteristics to benefit the performance of integrated circuits.

## 1. Introduction

According to Moore’s Law and Dennard’s Law, planar metal–oxide–semiconductor field-effect transistors (MOSFETs) should be scaled down over a long period of time. As the MOSFETs size shrinks, the short channel effect (SCE) becomes increasingly prominent, leading to the emergence of fin field-effect transistors (FinFETs). The FinFET structure, introduced in the 20 nm technology node to mitigate the side effects of the SCE in semiconductor devices, has been successfully applied to the 5 nm technology node. The fin channels have become thinner to improve the gate control capability, which becomes more severe as the size is further reduced. In addition, the height of the fins has gradually increased to allow for a larger effective width in the same area. However, these methods of strengthening FinFET structures are also approaching their limits. Thus, NS-GAA devices are some of the most promising candidates after FinFETs for 3 nm process nodes and for aggressive CMOS downscaling [1,2,3,4] because they offer excellent short channel control, superior electrostatics and optimized power consumption compared to FinFETs [5].

In addition to the areas mentioned above, NS-GAA transistors have a wider effective width in the same footprint, resulting in one of the unique features of NS-GAA transistors compared to FinFETs: the “fat-fin” effect. A fat fin is created by process non-idealities, causing an increased off-state leakage current in the bulk region below the bottom Si nanosheet. Sub-channel leakage is of great concern in scaled CMOS technologies and suppression of the parasitic channel in NS-GAA transistors is extremely challenging and much more complex than in FinFETs. There are various approaches being discussed to address this issue, including (i) a heavily doped substrate, which is referred to as a PTS module [6,7,8], and (ii) inserting an insulating dielectric layer for bottom isolation, which is called a BDI module and includes a dielectric layer inserted partially at the bottom of the gate or S/D [9] or a dielectric layer inserted at the bottom of both the gate and S/D [10,11].

In a general PTS structure, a P-type heavily doped substrate for NMOSs and an N-type heavily doped substrate for PMOSs are adopted to form an ultra-thin depletion layer between the S/D and the heavily doped fat fin, thus minimizing the consumption of the length of the fat fin to inhibit sub-channel leakage. However, at the same time, this may lead to a band-to-band tunneling (BTBT) current from the drain to the substrate P-N junction [6,12,13]. In addition, process volatility also affects the electrical properties of PTS structures, for example, some studies have investigated the effect of leakage in PTS structures caused by different depths of the S/D recess [11,14].

BDI modules, such as full BDI or partial BDI modules, are studied to reduce the off-state current and exhibit excellent electrical characteristics by blocking the bottom parasitic channel leakage path [11]. In addition, a partial BDI module can be divided into dielectric isolation under the source and drain (S/D) region and under the gate region. A new buried oxide nanosheet GAA structure has been proposed for improving the leakage by locally inserting an oxide material only under the gate region [9]. However, one shortcoming of a partial BDI module is that it does not take into account the bottom parasitic capacitance and gate substrate leakage or drain substrate leakage compared to full BDI modules [9,14]. The latest report on full BDI presented a thermal oxidation method to form a totally isolated dielectric layer under the bottom Si nanosheet [15,16]. On the one hand, the thermal oxidation time is strongly related to the channel width, so the oxidation process is difficult to control. Moreover, a high temperature process would result in fast atom diffusion in stacked Si/SiGe layers and the abrupt interfaces between the stacked Si/SiGe layers would be destroyed, which would disrupt the NS-GAA device’s structure and morphology and the quality of the formed suspended Si nanosheet channels [17,18,19]. On the other hand, preparing full BDI modules before S/D epitaxy, i.e., Full BDI_First, limits the ability to boost the mobility of NS-GAA devices, especially PMOSs [10]. The original, non-high-stress engineering based on a full BDI is unfavorable for hole mobility because the SiGe S/D region obtained by epitaxial growth on the dielectric materials contains more defects [20,21].

To tackle the issues mentioned above, in this paper, we demonstrated a novel S/D-first full BDI scheme that introduces a total isolation layer underneath the S/D and gate regions. The innovative fabrication scheme proposes full BDI formation after S/D epitaxy to circumvent the difficulty of introducing stress engineering in the full BDI formation before S/D epitaxy. In addition, the proposed Full BDI_Last scheme flow is compatible with the main fabrication flow of the NS-GAA transistor fabrication and provides a large window for process fluctuations, such as the thickness of the S/D recess. Compared with PTS engineering, the proposed full BDI module shows the benefits of significantly suppressing the parasitic channel, reducing the capacitance of gate and optimizing the performance explained by using 3D-TCAD. Moreover, the effects of a PTS, Full BDI_First and Full BDI_Last fabricated by the novel scheme on the operating speed and power consumption characteristics of a basic inverter RO are simulated separately.

## 2. Device Structure and Simulation Methodology

### 2.1. Device Structure

**Figure 1:**
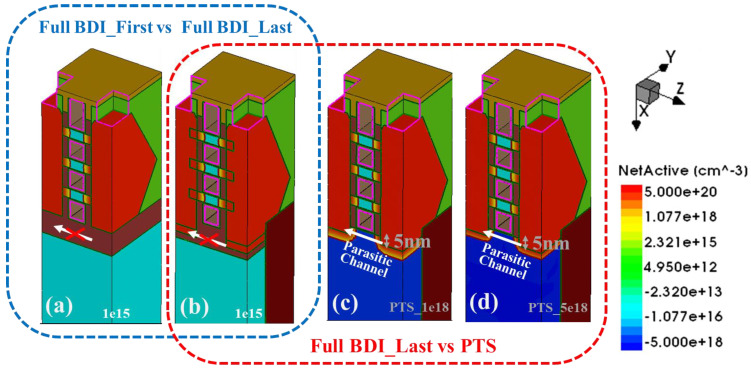
NS-GAA device structure with (**a**) Full BDI_First structure [11], (**b**) Full BDI_Last module, (**c**) PTS_1e18 module and (**d**) PTS_5e18 module.

Figure 1 shows the full BDI proposed in this study and two doping concentrations in the conventional PTS structure. The full BDI structure has a buried oxide below the S/D and gate regions. The basic parameters of the devices are designed with reference to the 3 nm technology nodes of the International Roadmap for Devices and Systems (IRDS) [22]. Among the geometrical parameters, the gate length was set to 12 nm and the Si nanosheet width and Si nanosheet thickness were fixed at 39 nm (close to the footprint of FinFET [23]) and 6 nm, respectively. Taking an N-type NS-GAA structure with Full BDI_Last as an example, the boron concentration of the substrate was 1e15 cm^−3^ and the depth of the S/D recess was set to 5 nm, as shown in Figure 1b, the same as Full BDI_First (Figure 1a). The excess S/D recess depth is strongly related to the bottom leakage path in NS-GAA devices, which represents the process volatility during S/D etching [24]. When comparing full BDI and PTS modules, the parameters of the PTS, which have also been primarily evaluated, include substrate doping concentrations of 1e18 cm^−3^ and 5e18 cm^−3^ and an S/D recess depth of 5 nm, as shown in Figure 1c,d. The aim of setting these parameters is to focus on the impact of process variation on the NS-GAA device. The thickness of the inner spacer in the above three device structures is set to 4 nm. Table 1 lists the structural parameters of the NS-GAA device with two kinds of full BDI and two doping concentrations in PTS modules, respectively.

### 2.2. Process Flow

**Figure 2:**
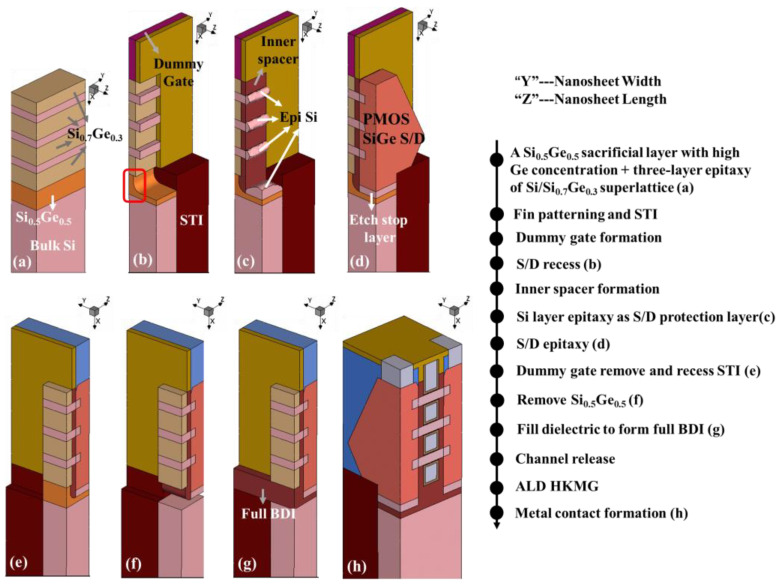
Cross-section of the key fabrication steps of a P-type NS-GAA device with the novel Full BDI_Last scheme.

A simplified 3D process simulation has been carried out based on the Sentaurus Process TCAD tool to verify the feasibility of this innovative Full BDI_Last fabrication scheme, as shown in Figure 2 (taking PMOS as an example). NS-GAA devices were fabricated on a Si substrate. A schematic of the key steps is demonstrated in Figure 2a–h, in which the channel length is along the *X*-axis direction. To implement Full BDI_Last engineering, Si_0.5_Ge_0.5_ with high Ge concentration was epitaxially grown first as a sacrificial layer for full BDI before the three-layer stacked Si/Si_0.7_Ge_0.3_ was grown. One particular note is that the thickness of the sacrificial Si_0.5_Ge_0.5_ layer for full BDI was 15 nm to be below the critical thickness for plastic relaxation [25], as shown in Figure 2a. Since the concentration of the sacrificial Si_0.5_Ge_0.5_ layer for full BDI is higher compared to three-layer stacked Si_0.7_Ge_0.3_, it will etch faster when the inner spacer is prepared, forming a groove circled by the red box in Figure 2b. Based the researched literature [26], the selective etching ratio of Si_0.7_Ge_0.3_ to Si_0.5_Ge_0.5_ can reach 1:1.5, which means that when the inner spacer is prepared to etch a 4 nm Si_0.7_Ge_0.3_ layer, the remaining thickness of the Si_0.5_Ge_0.5_ sacrificial layer at the bottom of the S/D will be at least 4 nm, which means the thickness of the BDI layer at the bottom of the S/D is at least 4 nm. The thickness of the BDI layer at the bottom of the gate remains at 15 nm. Then, a Si layer was epitaxially grown before the S/D epitaxial growth presented in Figure 2c, the aim of which is to prevent damage to the SiGe S/D region of the PMOS when the sacrificial Si_0.5_Ge_0.5_ layer is removed in the subsequent steps. STI in the channel width direction was performed to etch down a bit (see Figure 2e) and a side of the SiGe layer was exposed to facilitate the subsequent sacrificial Si_0.5_Ge_0.5_ layer etching, which is the most critical step in Full BDI_Last fabrication, as shown in Figure 2f. Additionally, Si_0.7_Ge_0.3_ was damaged a little (about 0.5–1 nm) according to the high selective etch ratio (at least 1:20) of Si_0.7_Ge_0.3_ to Si_0.5_Ge_0.5_ [27]. Figure 2g shows that the dielectric is filled into the original sacrificial Si_0.5_Ge_0.5_ layer to form the full BDI structure. As shown in Figure 2h, there is no conductive path under the S/D and gate regions. At the same time, the proposed Full BDI_Last scheme flow is compatible with the main process flow of NS-GAA transistor fabrication and provides a large window for process fluctuations, such as the thickness of the S/D recess.

### 2.3. Simulation Methodology

Synopsys Sentaurus TCAD with advanced physical models [28] was used to calibrate the electrical characteristics of the 3 nm node NS-GAA device. For an accurate analysis of nanoscale devices, a self-consistent calculation was used based on the drift-diffusion (DD) transport equation combined with the Poisson and carrier continuity equations. Ballistic mobility, high-field saturation models, Auger, Shockley–Read–Hall (SRH) and Hurkx BTBT models were also included [29]. Moreover, a thin-layer model and an inversion and accumulation layer (IAL) mobility model were utilized to account for the impurities, phonons, surface roughness and thin-layer-related scattering [30,31]. The Slotboom bandgap narrowing model was included to consider doping-dependent bandgap changes [32].

**Figure 3:**
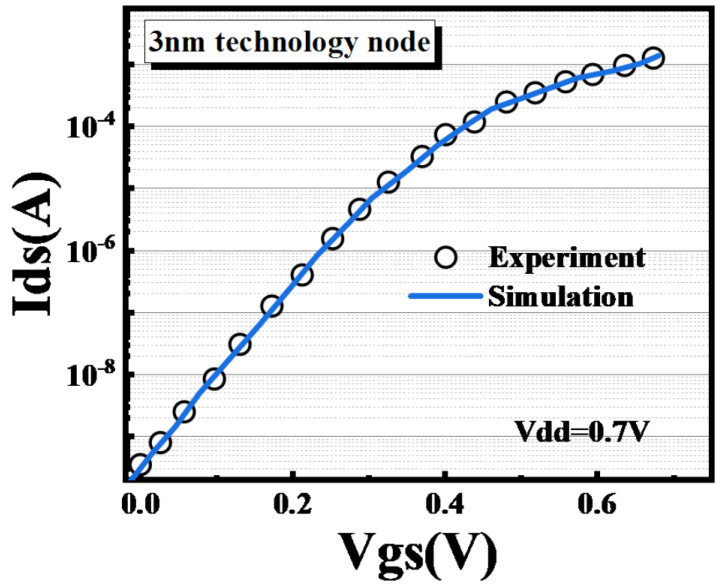
Ids versus Vgs calibration result based on the measurements of a fabricated 3 nm N-type GAA device, Experiment [33].

The calibration was conducted with key process parameters acquired from the experimental reports and the device’s geometrical dimensions were set according to SEM and TEM analysis information [33]. Additionally, the physical mode parameters in TCAD simulations were tuned to fit the experimental results to ensure the accuracy of the conclusions. Figure 3 shows the comparison of the simulated and measured transfer characteristics from the IBM 3 nm node GAA device data to succinctly prove the accuracy of the physical model parameters [34]. It is reasonable to implement the above-mentioned physical models as the simulated values fit well with the experimental measured values.

## 3. Results and Discussion

**Figure 4:**
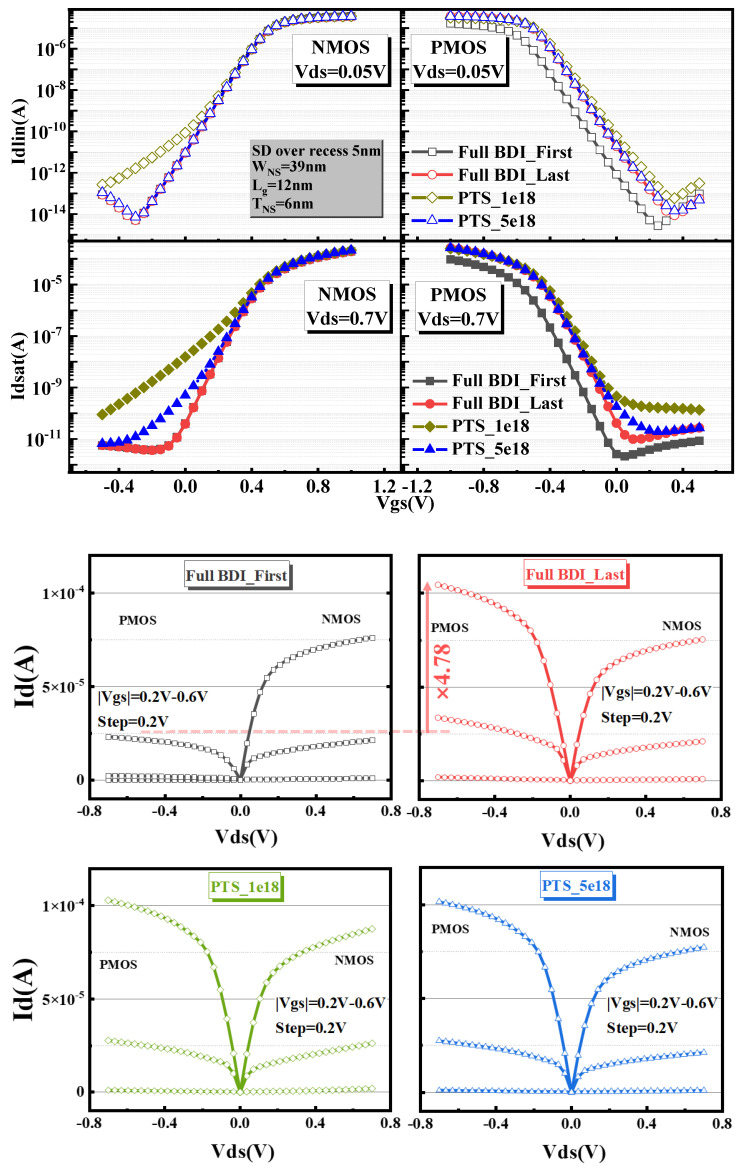
Id–Vg and Id–Vd characteristics for different full BDI schemes and different PTS degrees, corresponding to the structures in Figure 1a–d.

Figure 4 demonstrates the transfer curves of Full BDI_First and Full BDI_Last in n-type and p-type NS-GAA devices. In addition, a PTS module was also included in the Sentaurus Device TCAD simulation. The NS-GAA device structures with an S/D recess depth of 5 nm and substrate doping concentrations of 1e18 and 5e18 were envisaged in the Sentaurus Device electrical simulation. Figure 4 separately shows I–V characteristics for both n-type and p-type NS-GAA devices with a gate length of 12 nm with full BDI and a PTS module in the liner and saturated regions. It is not hard to observe that both n-type and p-type NS-GAA devices with Full BDI_Last show more outstanding electro-static behavior compared to PTSs with an S/D recess depth of 5 nm.

**Figure 5:**
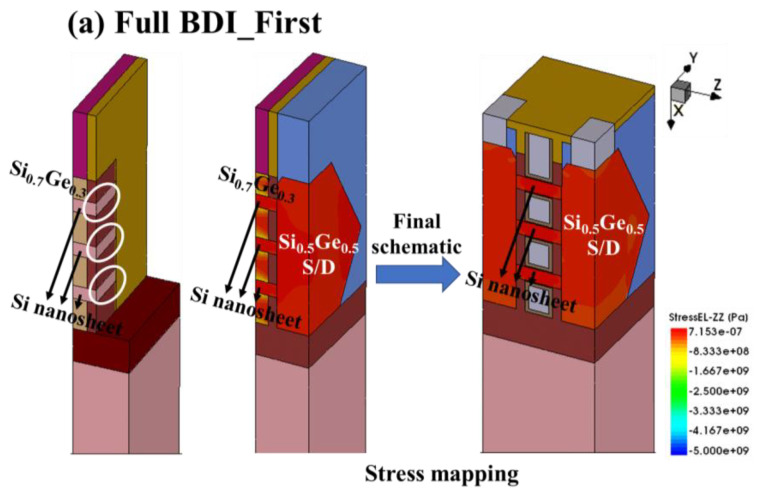

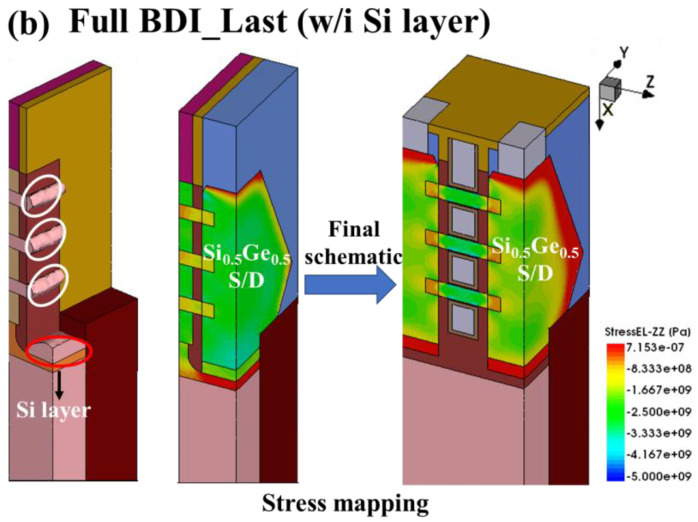
Comparison of the starting surface of S/D epitaxial growth and compressive stress mapping between Full BDI_Last and Full BDI_First of a P-type NS-GAA device.

It should also be noted that the Id–Vg electrical curves of the n-type NS-GAA device with Full BDI_First and Full BDI_Last structures overlap almost exactly, while for the PMOS, the Id–Vd curve extracted from Full BDI_Last shows a very significant improvement, with a 4.78 times higher drive current than that of Full BDI_First. This indicates that the stress engineering of Full BDI_Last proposed in the paper is more superior compared with Full BDI_First and thus the preparation process of Full BDI_Last is favorable for the driving capability of holes and can compensate for the balance between NMOS and PMOS. The specific reason is that the S/D epitaxial growth onset surfaces of Full BDI_First and Full BDI_Last are not the same. For Full BDI_First, the S/D epitaxial starting surface is the position circled in white in Figure 5a, i.e., the side of the three nanosheets, while for Full BDI_Last, the S/D epitaxial starting surface has an important bottom Si layer, circled by a red box in Figure 5b. Finally, the Sentaurus TCAD simulation shows that there is almost no compressive stress in the Si_0.5_Ge_0.5_ S/D region of Full BDI_First, leading to no compressive stress in the final Si nanosheet (Figure 5a). The compressive stress distribution of Full BDI_Last shows that the Si_0.5_Ge_0.5_ S/D has a high compressive stress and the final Si nanosheet has a high compressive stress, which is beneficial to hole mobility, as shown in Figure 5b.

**Figure 6:**
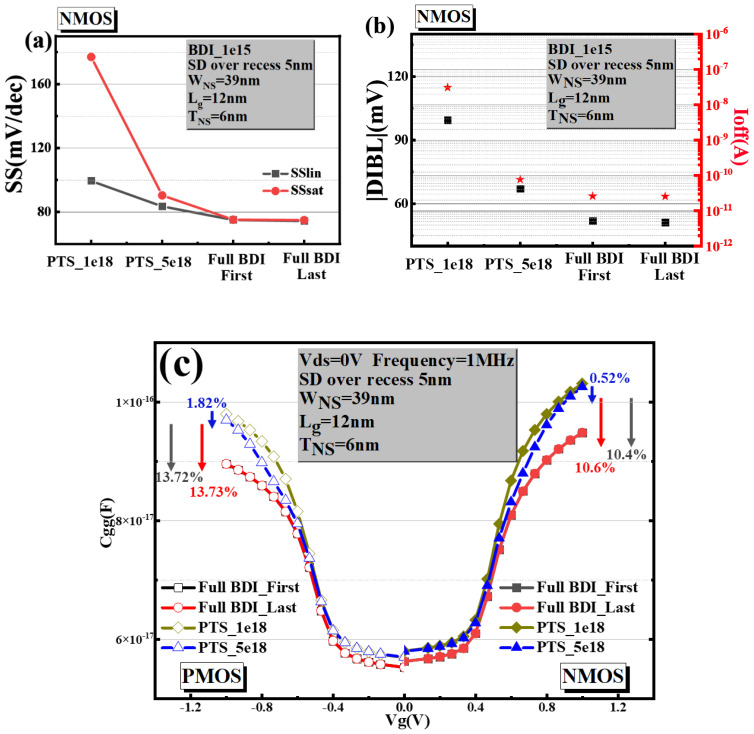
Comparisons of (**a**) SS, (**b**) DIBL and Ioff and (**c**) Cgg mapping among Full BDI_Last, Full BDI_First, PTS_1e18 and PTS_5e18 NS-GAA devices.

A comparison between the PTS module and the novel Full BDI_Last module was performed on the basis of the off-state leakage current, short channel effects and the effective capacitance (Ceff). Figure 6a,b shows the sub-threshold slope (SS), the drain-induced barrier lowering (DIBL) and the leakage current in the off state of full BDI and PTS modules from Sdevice simulation for the n-type NS-GAA device. These figures highlight that nanosheet structures with a gate length of 12 nm and a full BDI structure have obviously advantageous SS, DIBL and Ioff values compared with PTS_1e18 and PTS_5e18 on nanosheets with a gate length of 12 nm. Meanwhile, both the n-type and p-type NS-GAA devices with full BDI overcome the degradation of electrical characteristics caused by the parasitic sub-channel, which is because the full BDI compared to the PTS module cuts off the leakage current modes of the parasitic sub-channel, as shown by the blue arrow in Figure 1a–d, which degrades the electro-statics. Moreover, Full BDI has an intense immunity to non-ideal process variations [11], such as random dopant fluctuation (RDF), because of the heavily doped PTS, the significant S/D dopant diffusion into parasitic sub-channels due to the thermal budget and the S/D recess at extremely scaled gate length designs. The performance of the PTS module improved with an increase in the dopant level due to the degree of inhibition caused by the parasitic channel increase. However, the RDF effect in the PTS module increases significantly with the increasing concentration of the substrate. Since the NMOS electrical parameters are basically the same for both full BDI approaches, only the percentage degradation data of Full BDI_Last compared to PTS_1e18 and PTS_5e18 for different performance parameters are summarized in Table 2.

In addition to a higher leakage current and other performance indicators, the parasitic sub-channel will contribute to an aggravated Cgg, which will reduce NS-GAA device performance. Figure 6c show the Sdevice simulation result of gate capacitance in n-type and p-type NS-GAA devices that will be affected by introducing different PTS doping concentrations. The gate capacitance of the NS-GAA device with full BDI is the most dominant, which helps to reduce the parasitic capacitance which translates to a performance and RC delay boost. Taking PTS_1e18 as a basis, the gate capacitances of n-type and p-type NS-GAA devices with full BDI are reduced by 10.6% and 13.73%. The gate capacitance of n-type and p-type stacked Si nanosheets with a PTS module are obviously larger compared those with a full BDI module because of the formation of a narrow PN junction due to high substrate doping.

Circuit operation characteristics affect not only direct current (DC) I–V characteristics, but also alternating current (AC) C–V characteristics. The BSIM-CMG model library [35], which can accurately describe the I–V and C–V characteristics of NS-GAA devices according to the full BDI and PTS doping process, was also simulated in Sentaurus Device TCAD.

**Figure 7:**
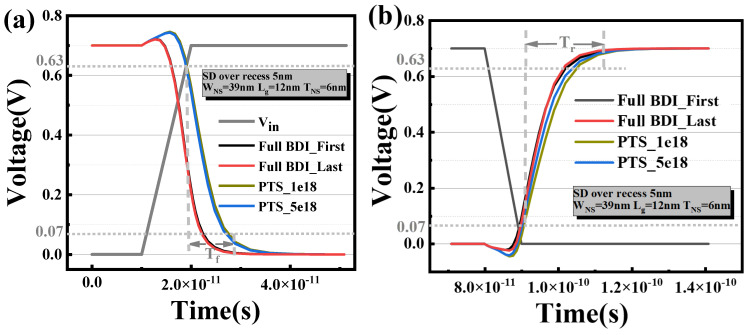

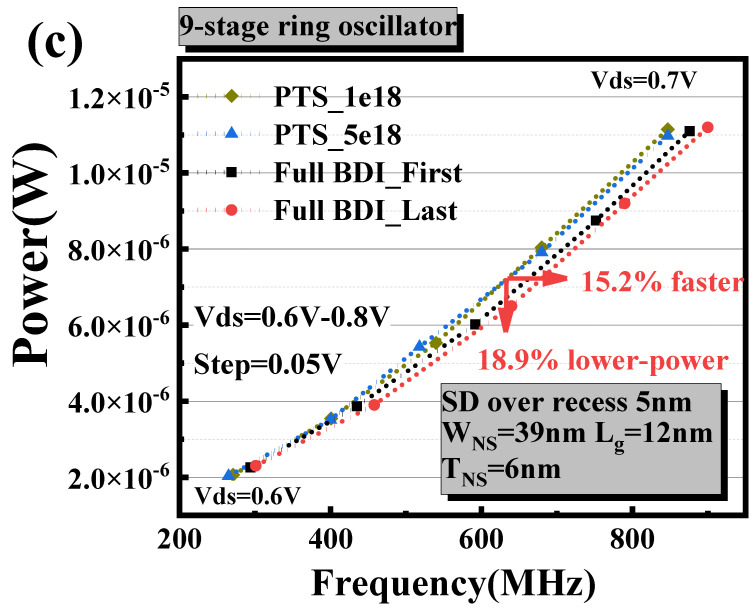
Simulation results of inverter and nine-stage RO for (**a**) rise delay, (**b**) fall delay and (**c**) PP (power and performance) of the NS-GAA device.

Figure 7a,b shows the fall delay and rise delay of inverters composed of devices prepared by PTSs and full BDI, respectively. It is clear that the delay of the inverter with a full BDI composition is the smallest for both rise and fall delays. Compared to Full BDI_First, Full BDI_Last demonstrates a slight improvement in the electrical performance of the inverter. The RO used for this benchmark is an inverter chain consisting of nine stages. Figure 7c shows the simulation results of the active power and operating frequency of the nine-stage RO while linearly changing the supply voltage, Vds (Vds = 0.6 V–0.8 V, step = 0.05 V). The simulation curves of the two PTS modules with different doping concentrations almost coincide. Compared to the PTS module and Full BDI_First module, it can be seen that the flip speed of the full BDI fabricated by the novel scheme increased by 15.2% and 6.2% under the iso-power condition, and the consumed power reduced by 18.9% and 6.8% under the iso-speed condition separately.

## 4. Conclusions

In this work, the formation of a Full BDI_Last device and its electrical properties have been discussed based on Sentaurus TCAD simulations. Full BDI_Last was constructed by a novel fabrication flow combining the S/D-first scheme and introducing a sacrificial Si_0.5_Ge_0.5_ layer underneath the S/D and gate region, aiming to inhibit parasitic channels in the Si substrate. After the S/D epitaxial growth, the bottom sacrificial Si_0.5_Ge_0.5_ layer was completely removed and filled with a dielectric material to form a full BDI structure. In particular, employing a Si layer as the etch stop layer to protect the SiGe S/D region in the P-type NS-GAA device was completely feasible during direct removal of the Si_0.5_Ge_0.5_ layer.

On the one hand, the Full BDI_Last preparation process is compatible with the main fabrication flow of NS-GAA transistors. Most importantly, the Full BDI_Last module displayed great immunity to process volatilities such as the S/D recess and RDF due to a heavily doped PTS. Furthermore, the innovative process is beneficial for S/D epitaxial reinforced stress engineering because the S/D-first scheme provides a high-quality starting surface for S/D epitaxial growth compared with Full BDI_First. On the other hand, the full BDI module used in the NS-GAA device can potentially offer significant benefits over the PTS module. In terms of parasitic sub-channel leakage, the full BDI technique provides a solution for removing the parasitic sub-channel to control leakage and improves the electrical properties in all aspects, including reducing the parasitic capacitance compared to the PTS module. Moreover, it was verified that in an inverter RO, which is a logic circuit, it is possible to increase the speed by 15.2% and 6.2% under iso-power conditions and decrease the power by 18.9% and 6.8% under iso-speed conditions, compared to the PTS scheme and the Full BDI_First scheme, respectively. The novel full BDI scheme present in the NS-GAA device provides some guidance for the subsequent preparation of high-performance circuits.

## Figures and Tables

**Table 1:** Physical parameters of the NS-GAA device in the Sentaurus TCAD simulation.

Parasitic Channel Isolation Method	Nanosheet Length	Nanosheet Width	Nanosheet Thickness	S/D Recess	Substrate Doping Concentration
Full BDI_First	12 nm	39 nm	6 nm	5 nm	1e15 cm^−3^
Full BDI_Last					
PTS	12 nm	39 nm	6 nm	5 nm	1e18 cm^−3^
					5e18 cm^−3^

**Table 2:** The percentage reduction of different performance parameters for Full BDI_Last compared to PTS_1e18 and PTS_5e18.

Parameter	PTS_1e18	PTS_5e18
SSlin	25.2%	11.1%
SSsat	70.2%	17.5%
|DIBL|	58.4%	23.7%
Log|Ioff|	48.5%	15.4%

## Data Availability

The data that support the findings of this study are available from the corresponding author upon reasonable request.
